# A Case Report on Generalised Muscle Spasms After Ornamental Tarantula Bites That Responded Well to Intravenous Calcium Gluconate Treatment

**DOI:** 10.7759/cureus.43074

**Published:** 2023-08-07

**Authors:** Ramanathan Ramesh, Arulmoly Kanagasingam, Uthayakumar Anushanth, Aruna Gunasinghe, Navaneethakrishnan Suganthan

**Affiliations:** 1 General Medicine, Teaching Hospital Batticaloa, Batticaloa, LKA; 2 Medicine, Teaching Hospital Batticaloa, Batticaloa, LKA; 3 Medicine, University of Jaffna, Jaffna, LKA; 4 University Medical Unit, Teaching Hospital, Jaffna, Jaffna, LKA

**Keywords:** calcium gluconate, muscle spasms, spider bite, ornamental tarantula, poecilotheria fasciata

## Abstract

Here, we present a case of a 55-year-old male, who was admitted with a spider bite, which caused swelling of the hand and painful muscle spasms along with palpitations. The patient made a complete recovery after the administration of intravenous calcium gluconate, followed by oral calcium supplements. Although no specific treatment exists in Sri Lanka for spider bites, calcium supplements can be beneficial for Sri Lankan ornamental tarantula (Poecilotheria fasciata) bites.

## Introduction

Numerous studies have shown that there are in excess of 41,000 kinds of spiders present in the world [1]. Data show that only a handful of species are venomous, and spiders are part of Arthropoda and belong to the class Arachnida [2]. Most spiders have venomous glands, but they rarely bite because their fangs are small and do not penetrate the skin [3]. Australia and Latin American countries are home to a variety of venomous spiders, including the brown recluse spider (Loxosceles spp.), black widow spider (Latrodectus spp.), funnel web spider (Atrax and Hadronyche spp.), and ornamental tarantula spider (Poecilotheria), all of which have highly toxic venom. The Sinhala language calls the most venomous spiders "Divimakuluwa," while Tamil people name them "Nachchu Silanthi or Pulimuga Silanthi," in Sri Lanka. Incidents of venomous spider bites have been officially recorded in Sri Lanka. Poecilotheria fasciata is called a Ceylon-hunting spider. It is a hairy spider that is about 15-20 cm in length. So far, there were no reported fatalities because of Ceylon-hunting spider bites in Sri Lanka. They live in trees and are usually seen in the dry areas of the country. The bites of Ceylon-hunting spiders are as feared by people in villages as that of venomous snakes. Spider venom has several chemical components. Among them, proteolytic enzymes, serotonin, and histamine are significant. A spider bite can lead to intense local pain and muscle spasms [4]. Rarely, envenomation may cause systemic effects, such as neurotoxicity, acute renal failure, pulmonary haemorrhage, intravascular haemolysis, and acute pancreatitis. Venom neurotoxin can interfere with voltage-gated potassium channels by interacting with the voltage-sensing domain of the potassium channel, which makes with the excitability of the skeletal muscle fibres, leading to painful muscle spasms [5]. There is no antivenom available in Sri Lanka in the event of a bite from Ceylon-hunting spiders. Intravenous calcium gluconate can be used effectively in Sri Lanka for muscle spasms caused by spider bites [5]. We present a case of a 55-year-old male, with a spider bite from an ornamental tarantula (Poecilotheria fasciata), who developed generalized muscle spasms and well-responded to intravenous calcium gluconate treatment.

## Case presentation

A previously unevaluated 55-year-old male was taken to the hospital due to a spider bite while cutting grass in the garden. A spider had bitten on the palmar aspect of his right hand. The patient was admitted with a complaint of painful swelling on the right side of his hand. The spider was taken to the hospital and identified as a Poecilotheria fasciata. The patient's blood investigation is shown in Table 1.

**Table 1:** His blood investigation results WBC-white blood cells, Hb-Haemoglobin, AST-aspartate amino-transferase, ALT- alanine amino-transferase, ALP-alkaline phosphatase, GGT- gamma glutamyl transferase, CPK-creatinine phosphokinase

Investigation	Results	Normal Range
WBC (microL)	12,910	4,000-11,000
Hb (g/dL)	14.6	14-17
Platelets (micro/L)	272,000	150,000-450,000
Sodium (mmol/L)	136	136-145
Potassium (mmol/L)	3.7	3.5-4.5
Calcium (mmol/L)	2.1	2.2-2.6
Phosphate (mmol/L)	1.4	0.97-1.45
Magnesium (mmol/L)	0.8	0.66-1.07
Creatinine (micromol/L)	70	62-115
Blood urea (mmol/L)	5	2.1-8.5
AST (U/L)	35	14-20
ALT (U/L)	43	10-40
ALP (U/L)	120	30-120
GGT (U/L)	136	5-40
Total protein (g/L)	56	60-80
Globulin (g/L)	21	20-35
Albumin (g/L)	35	35-55
CPK (mcg/L)	1,089	10-120

After 24 hours, the patient developed generalized painful muscle spasms. On initial assessment, we observed swelling in his right hand and generalized muscle twitches. The patient’s pulse rate was 94 beats/min. His blood pressure was 110/70 mmHg. Peripheral oxygen saturation was 100% on ambient air. Apart from visible generalized muscle spasms, the patient did not have any other neurological issues. The patient showed no signs of coagulopathy or acute kidney injury such as purpuric/ecchymotic rashes or other bleeding manifestations or reduced urine output or fluid overload signs.

The ECG revealed multiple ventricular ectopics. We have given adequate pain relief with regular paracetamol 1 g three times a day and chlorpheniramine 4 mg twice a day for his allergic symptoms. The arm was elevated to decrease the hand swelling. The patient was well-hydrated with 0.9% saline and 100cc per hour infusion, and the input and output were monitored to avoid acute kidney injury. Because the patient had frequent ventricular ectopics, he underwent ECG monitoring. Although the patient's serum calcium level was normal, he was administered an intravenous 10% calcium gluconate 10cc over 10 minutes, and the patient's muscle spasms well-responded. Following the initial dose of calcium gluconate, an oral calcium supplement was started. He has been discharged after 48 hours of hospital stay and was advised to take enough fluid and pay attention to his urine output. We carried out a review of him with a full blood count, renal function, CPK, and ECG one week later. The patient made a complete clinical recovery, and his repeated investigations were normal.

## Discussion

The clinical manifestation and treatment modalities are not well-documented in medical literature in Sri Lanka following the bites of ornamental tarantula (Poecilotheria fasciata), except for available few case reports [5]. Venomous species include the brown recluse spider, black widow spider, funnel web spider, and tarantula spider. Even though antivenoms to treat the bite of the brown recluse spider, black widow spider, and funnel web spider are available, none of those species is present in Sri Lanka. The pathophysiology of muscle spasms following ornamental tarantula bites is not yet well-studied. It is hypothesized that venom neurotoxins can directly influence the calcium and sodium channels of the skeletal muscles, resulting in muscle spasms. In addition, venom neurotoxin can interfere with voltage-gated potassium channels by interacting with the voltage sensing domain of the potassium channel, which makes with the excitability of the skeletal muscle fibers, leading to painful spasms. Muscle spasms are refractory to supportive management such as hydration and simple analgesics, but it has been well-responded to intravenous calcium [6]. So intravenous calcium can be used as the first-line treatment modality for painful skeletal muscle spasms following the bite of the ornamental tarantula. Although intravenous calcium is considered a first-line treatment for ornamental tarantula bites, it is ineffective for pain as compared with the combination of intravenous opioids and benzodiazepine [7]. Clinical manifestations following a spider bite depend on the species of spider and the amount of venom injected. A spider bite can cause a localized reaction, which appears as a swollen area at the bite site and pain. Despite local manifestations, it causes systemic manifestations as well. Abdominal pain and vomiting are two common systemic issues that are related to the gastrointestinal system. The renal, pulmonary, and cardiovascular systems are affected less frequently.

## Conclusions

The brown recluse spider (Loxosceles spp.), black widow spider (Latrodectus spp.), funnel web spider (Atrax and Hadronyche spp.), and tarantula spider (Poecilotheria fasciata) are considered as venomous species. Venom contains a variety of pharmacological substances. Histamine and serotonin are the two substances identified. Poecilotheria fasciata envenomation can lead to skeletal muscle spasms, intravascular haemolysis, rhabdomyolysis, acute kidney injury neurotoxicity, and cardiac toxicity. However, in our patient, it only causes skeletal muscle spasms, other than local swelling and pain, and it responded well to intravenous calcium gluconate.
